# Respiratory and sleep characteristics based on frequency distribution of craniofacial skeletal patterns in Korean adult patients with obstructive sleep apnea

**DOI:** 10.1371/journal.pone.0236284

**Published:** 2020-07-20

**Authors:** Su-Jung Kim, Hyo-Won Ahn, Kyoung Jin Hwang, Sung-Wan Kim

**Affiliations:** 1 Department of Orthodontics, Kyung Hee University School of Dentistry, Seoul, Korea; 2 Department of Neurology, Kyung Hee University School of Medicine, Seoul, Korea; 3 Department of Otorhinolaryngology-Head and Neck Surgery, Kyung Hee University School of Medicine, Seoul, Korea; AUSL della Romagna, ITALY

## Abstract

**Objective:**

To investigate the frequency distribution of various craniofacial skeletal patterns in a large Korean adult obstructive sleep apnea (OSA) population, and to find a relationship between craniofacial risks and respiratory and sleep characteristics.

**Methods:**

A total of 1226 OSA patients (mean age of 44.9±13.3 years) were included in this retrospective cross-sectional study. All subjects were evaluated for gender and age using fourteen polysomnographic, five cephalometric, two comorbid variables, and three self-reported indexes. Frequency analysis was used to screen the distribution of main skeletal patterns and subtypes. Intergroup comparisons were performed using independent t-test, chi-square test or analysis of variance. Univariable regression analysis was done to find a relationship between skeletal risks and OSA characteristics.

**Results:**

The frequency distribution of skeletal patterns was as follows: sagittally 57.2%, 32.3%, and 10.5% of Class II, Class I, and Cass III; vertically 54.0%, 26.7%, and 19.3% of hyperdivergent, normodivergent, and hypodivergent type, respectively. Polysomnographic, symptomatic, and comorbid variables showed no differences among patients with different skeletal patterns. Conversely, skeletal variables showed no differences according to OSA severity. The prevalence of highly risky skeletal pattern of hyperdivergent Class II was more likely to be females (OR 4.52, *P* < .01) and less obese (OR 3.21, *P* < .01), irrelevant to OSA and sleep characteristics.

**Conclusion:**

Characteristic frequency distributions of skeletal patterns and subtypes were observed in adult OSA patients however, no statistical association was found between the skeletal patterns and OSA characteristics due to the large interindividual variation.

## Introduction

Obstructive sleep apnea (OSA), defined as repetitive episodes of upper airway obstruction during sleep, is generally promoted by structural and functional abnormalities in a complicated way [1]. OSA is characterized by a heterogeneity in predisposing factors, pathophysiologic mechanisms, clinical presentations, long-term health outcomes, and treatment responses [2]. Previous studies have attempted to suggest clinically available OSA phenotypes by classifying OSA into more homogeneous subtypes [3–6], however, no clear phenotype encompassing various measurable features such as signs, symptoms, demographic factors, polysomnographic and morphometric factors, and comorbidities, has been obtained so far. Recently, the novel concept of phenotypic causes of adult OSA has been established in pursuit of phenotype-based precision therapeutic approach: anatomically collapsible upper airway, inadequate responsiveness of upper airway dilator muscles, premature arousal to airway narrowing, and oversensitive ventilator control system [7]. Out of these, anatomically collapsible upper airway is the key cause of OSA patients, although one or more non-anatomic pathophysiologic traits are combined in many patients [8]. Therefore, the importance of craniofacial anatomical evaluation and management is being highlighted to recognize and treat the patients who belong to the craniofacial anatomical phenotype of OSA.

Anatomically collapsible upper airway is mostly attributed to obesity, enlarged upper airway soft tissues, and skeletal deformity [9]. In particular, skeletal deformities predisposing to the airway collapsibility have been reported to include short cranial base length, acute cranial base flexure, mandibular retrognathism, maxillary hypoplasia, maxillary transverse constriction with or without nasal cavity obstruction, hyperdivergent vertical pattern with steep occlusal and mandibular planes, which result in pharyngeal narrowing and lengthening with downward displacement of the hyoid, and backward displacement of the tongue base and soft palate [10,11]. Currently, a meta-analysis of 25 studies [12] reported stronger correlation between OSA and vertical skeletal discrepancy such as excessive lower facial height and inferiorly displaced hyoid, than sagittal skeletal deformity like depressed maxilla or retrognathic small mandible which showed large heterogeneity among studies. Most of the previous studies described the common skeletal characteristics of OSA patients based on the mean value of each skeletal parameter from all subjects [13,14], however, there is no large population study investigating the distribution of various types of skeletal patterns in adult OSA patients matching with sleep characteristics.

Ethnic difference in craniofacial morphology may influence the risk for OSA across racial groups [15]. Interracial comparative studies revealed that Asian OSA populations primarily displayed features of craniofacial skeletal restriction. African Americans exhibited higher obesity and enlarged upper airway soft tissues, while Caucasians showed evidence of both skeletal and soft tissue abnormalities [16,17]. A more recent study summarized that craniofacial restriction may be more important in OSA risk in Chinese than in Caucasian patients [15], and Chinese patients appeared to present more severe OSA than Caucasians when the body mass index (BMI) was matched at the same level [18,19]. As for Koreans, we had previously identified and characterized three clusters of OSA patients by integrating craniofacial features into sleep characteristics [20]. Of all Korean OSA adults, 61.4% had definite craniofacial risk factors like skeletal Class II with retrognathic mandibles and hyperdivergent vertical patterns, with or without nonanatomic causative factors, however 38.6% of the patients proved not to have skeletal vulnerability. This raised a question of relative distribution of various skeletal subtypes in a large OSA population and its relationship with diverse aspects of OSA characteristics.

Understanding the craniofacial skeletal pattern in association with OSA presentations would advance clinician’s contribution to comprehensive OSA management. We aimed to investigate the frequency distribution of various skeletal patterns in a large Korean adult OSA population, and to find a relationship between craniofacial risks and OSA severity, sleep quality, symptoms, and comorbidity.

## Materials and methods

### Study subjects

**Fig 1:**
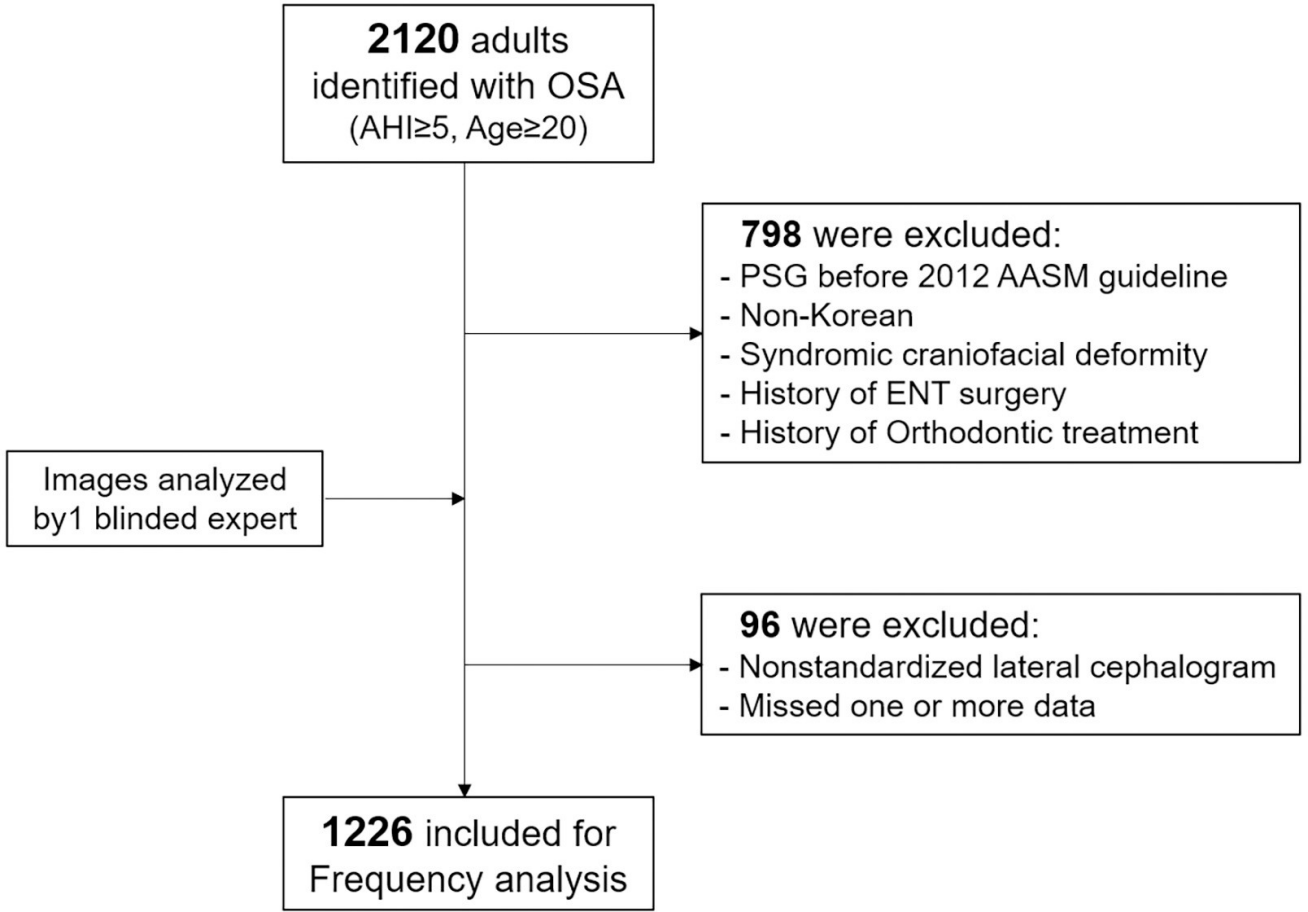
A flow diagram for sample selection.

We identified 2120 adult patients aged over 20 years who were diagnosed as OSA with apnea hypopnea index (AHI)≥5 events/hour at Kyung Hee University medical center in Seoul, Korea, between January 2013 and December 2019. The medical and dental charts of all patients were retrospectively reviewed to obtain the clinical information from interview, medical examination, medication history related to hypertension and diabetes mellitus, and dentofacial examination. The exclusion criteria were as follows: the patients with (1) Non-Korean; (2) history of upper airway surgery; (3) history of orthodontic treatment or orthognathic surgery; (4) syndromic craniofacial deformity; (5) non-standardized lateral cephalogram; (6) one or more missed record and poor quality of data; (7) progressed central apnea with serious comorbidity such as pulmonary or renal problem. A total of 1226 patients (mean age of 44.9 ± 13.3 years; from 20.0 to 78.5 years) were included in the analysis (Fig 1). This study was approved by the Institutional Review Board at Kyung Hee University Dental Hospital (KHD IRB 1811–2). The consent was not obtained because this study was retrospective and the data were analyzed anonymously.

### Polysomnography

A trained sleep technologist manually scored the breathing and sleep events from full standard in-laboratory polysomnography (PSG), following the standard from 2012 American Academy of Sleep Medicine manual [21]: overall AHI, AHI in a supine position (AHI_supine_), AHI in a rapid eye movement (REM) stage (AHI_REM_), AHI in a non-REM stage (AHI_NREM_), respiratory disturbance index (RDI), the lowest oxygen saturation (LSaO_2_), oxygen desaturation index (ODI), proportion of REM stage throughout sleep, sleep efficiency, arousal index, and wake period after sleep onset (WASO). The severity of OSA was basically defined as follows: 5 ≤ AHI < 15, mild; 15≤AHI<30, moderate; AHI ≥30, severe. The body mass index (BMI, kg/m^2^) indicated the degree of obesity following the reference in Korea: 18.5≤BMI<23, normal weight; 23≤BMI<25, overweight; BMI≥25, obesity [22].

In addition, the level of arousal threshold (ArTh) was calculated according to the following equation: ArTh = −65.39 + (0.06 × age) + (3.69 × gender [where male = 1, female = 0]) − (0.03 × BMI) − (0.11 × AHI) + (0.53 × Nadir SaO_2_) + (0.09 × % of the overall respiratory hypopneas) [23]. Low ArTh was defined as an overall ArTh less negative than -15 cm H_2_O and high ArTh more negative than -15 cm H_2_O.

### Symptoms and comorbidity

Representative subjective symptoms of excessive daytime sleepiness (EDS) were assessed by Epworth sleepiness scale (ESS): scores of 1~6, enough sleep; 7~8, average sleep with tendency of sleepiness; over 9, very sleepy state requiring specialist’s care [24]. In addition, Stanford sleepiness scale (SSS) was estimated for rating of alertness during the day: a score below 3 indicated serious sleep deprivation [25]. Self-reported insomnia severity index was obtained. Two comorbid conditions of hypertension and diabetes mellitus were examined based on the medication history taking and medical chart review.

### Cephalometric analysis

**Fig 2:**
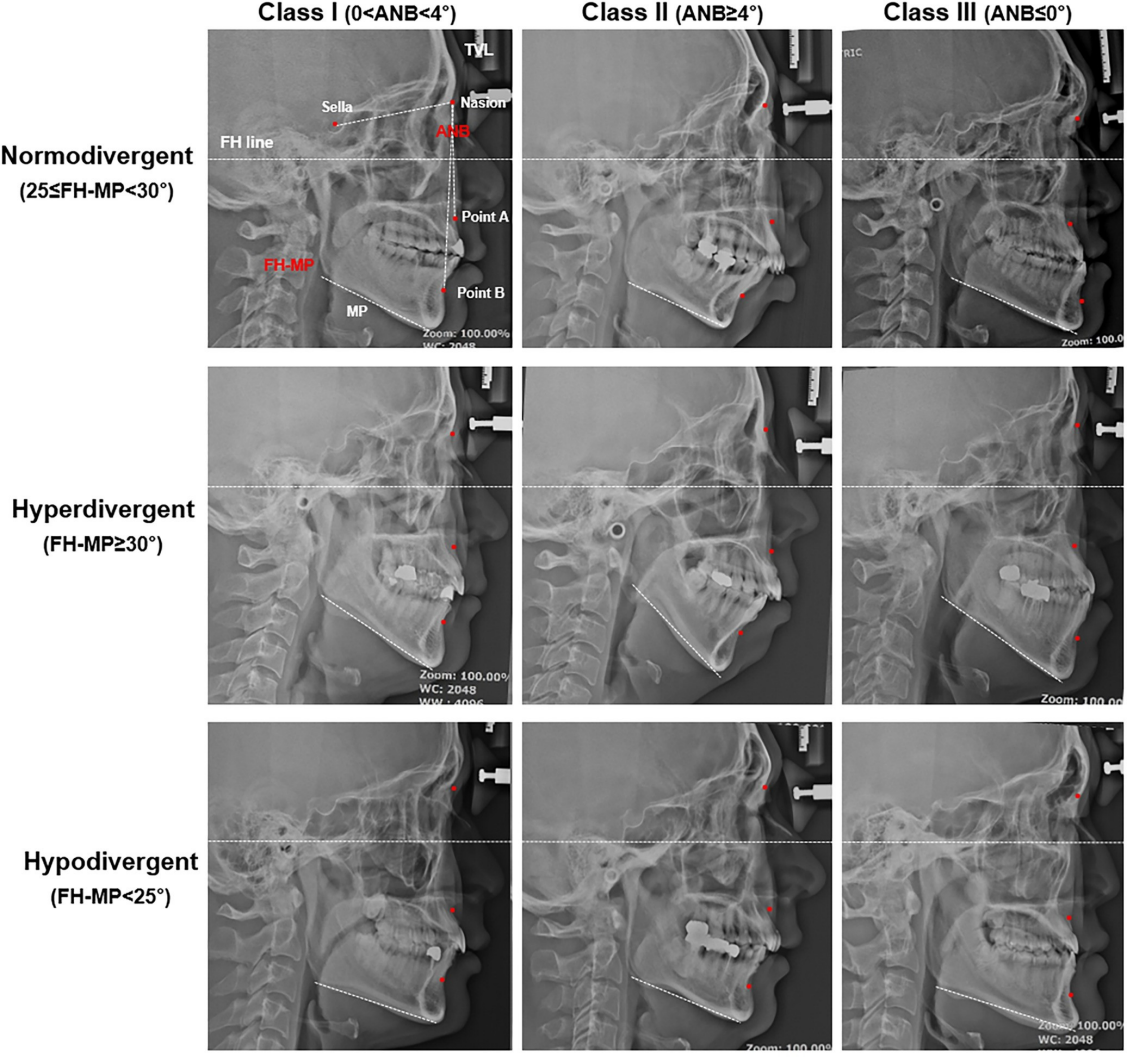
Lateral cephalometric classification of skeletal patterns for craniofacial phenotyping in adult OSA patients. Sagittal pattern was classified into Class I, Class II, and Class III according to ANB angle (Point A-Na-Point B), and vertical pattern was classified into normo-, hyper-, and hypodivergent pattern according to mandibular plane angle (FH-MP).

Standardized lateral cephalograms taken in a natural head posture at the end of expiration with normal breathing were analyzed by one expert orthodontist. To classify and subdivide the sagittal skeletal pattern, SNA, SNB, and ANB angles were measured: 0°<ANB<4°, Class I; ANB≥4°, Class II; ANB≤0°, Class III (Fig 2). To assort the vertical skeletal pattern, mandibular plane angle to the Frankfurt horizontal line (FH-MP) was measured: 25°≤FH-MP<30°, **normodivergent**; FH-MP≥30°, hyperdivergent; FH-MP<25°, hypodivergent pattern. The facial height ratio was calculated as posterior facial height (PFH) divided by anterior facial height (AFH). All variables were measured two times in 2 week-interval using a V-ceph program version 8.0 (OSSTEM IMPLANT Co., Seoul, Korea). The intra-class correlation coefficients (ICCs) calculated to assess intra-observer reliability for the repeated measurements ranged from 0.945 to 0.992. The mean value of two measurements was used for the analysis.

### Statistical analysis

Continuous variables were described with means ± standard deviations and compared using analysis of variance or independent *t*-test, based on the number of groups compared. Categorical variables were summarized using frequency and percentage and compared between groups using Pearson’s chi-square test. The distribution of sagittal and vertical skeletal classifications according to the gender and age groups were evaluated using frequency analysis. Unadjusted odds ratio (OR) and 95% confidence interval (CI) were estimated by univariable logistic regression analysis for selected variables. The dependent variable was set as skeletal Class II with hyperdivergent pattern which is a known highly risky skeletal factor [26–28]. *P*-values of <0.05 were considered statistically significant. All statistical analyses were performed with SPSS version 20 (SPSS Inc., Chicago, IL).

## Results

### Sample characteristics by gender and age

In total, 1226 Korean OSA patients were characterized as follows (Table 1): a mean BMI of 26.3 ± 3.6 kg/m^2^; AHI of 41.7 ± 22.2 events/hr; AI of 19.5 ± 21.2 events/hr; AHI_supine_ of 52.3 ± 21.7 events/hr; AHI_REM_ of 36.6 ± 15.2 events/hr; RDI of 43.8 ± 21.4 events/hr; ODI of 29.2 ± 28.9 events/hr; LSaO_2_ of 82.5 ± 7.9%; sleep efficiency of 88.1 ± 9.4%; REM stage of 18.1 ± 6.5%; arousal index of 43.5 ± 19.9 events/hr, and WASO of 10.8 ± 12.8%. The mean ArTh was -15.9 ± 7.9 cm H_2_O. The mean ESS, SSS, and insomnia severity index were 9.8 ± 5.0, 3.0 ± 1.2, 11.3 ± 6.1 respectively.

**Table 1:** Characteristics of subjects according to gender and age.

Variables		Total (n = 1226)			Gender							Age						
					Male (n = 983, 80.2%)			Female (n = 243, 19.2%)			*P*-value	20~50 years (n = 600, 48.9%)			50~80 years (n = 626, 51.3%)			*P*-value
Demographic	Age (years)	44.9	±	13.3	43.7	±	13.3	51.1	±	11.4	.013*	35.8	±	8.8	57.7	±	5.6	.000***
Polysomnographic	BMI (*kg/m*^*2*^)	26.3	±	3.6	27.6	±	3.6	25.0	±	3.4	.045*	26.5	±	4.0	26.2	±	3.8	.254
	AHI (*event/hr*)	41.7	±	22.2	42.6	±	22.0	33.4	±	19.1	.003**	39.1	±	23.0	43.3	±	20.0	.598
	AI (*event/hr*)	19.5	±	21.2	23.5	±	15.7	18.2	±	13.1	.043*	18.8	±	22.6	20.8	±	19.1	.955
	AHI_supine_ (*event/hr*)	2.3	±	21.7	53.1	±	21.1	42.2	±	14.3	.022*	50.7	±	12.8	56.3	±	13.0	.041*
	AHI_NREM_ (*event/hr*)	41.8	±	20.8	42.9	±	21.0	31.0	±	18.2	.044*	36.1	±	21.4	46.5	±	22.0	.045*
	AHI_REM_ (*event/hr*)	6.6	±	15.2	34.6	±	16.0	33.5	±	15.4	.055	37.2	±	17.0	34.1	±	20.0	.198
	RDI (*event/hr*)	43.8	±	21.4	45.6	±	21.1	37.0	±	21.6	.056	42.2	±	19.9	46.3	±	19.1	.711
	ODI (*event/hr*r)	29.2	±	28.9	31.0	±	29.8	21.7	±	22.1	.055	28.9	±	30.3	29.9	±	19.6	.555
	LSaO_2_ (*%*)	82.5	±	7.9	81.8	±	7.9	85.5	±	7.5	.015*	83.1	±	8.0	80.2	±	7.5	.005**
	Sleep efficiency (%)	88.1	±	9.4	87.3	±	8.1	91.6	±	10.2	.025*	90.3	±	8.0	86.6	±	7.3	.012*
	REM (*%*)	18.1	±	6.5	20.3	±	7.1	19.5	±	7.5	.252	18.8	±	22.6	18.1	±	6.2	.098
	Arousal index *(event/hr)*	43.5	±	19.9	42.6	±	20.1	46.5	±	18.5	.062	41.8	±	21.4	44.8	±	19.9	.056
	ArTh (*cm H*_*2*_*O*)	-15.9	±	7.9	-18.0	±	6.9	-12.4	±	5.7	.002**	-12.4	±	5.9	-17.2	±	6.4	.045*
Cephalometric	SNA (°)	82.6	±	2.5	82.9	±	2.3	81.9	±	2.0	.211	82.0	±	2.5	82.5	±	2.1	.233
	SNB (°)	78.3	±	3.0	78.8	±	3.2	76.9	±	2.3	.005**	78.6	±	2.2	78.3	±	2.1	.069
	ANB (°)	4.3	±	2.1	4.0	±	2.0	5.5	±	1.6	.001**	4.1	±	2.1	4.6	±	1.9	.156
	Mandibular plane to FH (°)	25.7	±	5.2	25.2	±	5.1	29.1	±	4.5	.001**	25.7	±	5.3	25.8	±	2.0	.378
	Facial height ratio (%)	66.5	±	5.2	69.2	±	5.1	63.1	±	4.5	.003**`	64.5	±	7.2	67.5	±	8.3	.265
Symptomatic	ESS (*score*)	9.8	±	5.0	10.2	±	6.2	9.3	±	5.1	.200	9.8	±	4.2	9.8	±	6.7	.865
	SSS (*score*)	3.0	±	1.2	3.7	±	2.0	2.9	±	1.6	.312	3.0	±	1.2	2.9	±	1.4	.198
	WASO (%)	10.8	±	12.8	11.5	±	12.2	13.8	±	14.5	.052	9.3	±	7.3	15.1	±	10.6	.015*
	Insomnia (*score*)	11.3	±	6.1	10.0	±	5.5	12.0	±	6.9	.102	11.5	±	5.5	11.3	±	5.7	.223
Comorbidity	Hypertension (*%*) ^§^	25.9			24.4			27.5			.347	13.7			41.4			.000***
	Diabetes (*%*) ^§^	6.8			7.1			5.8			.466	3.2			12.1			.002**

Independent t-test or chi-square test

(§) was performed:

*, P < .0.05

**, P < .0.01

***, P < .0.001. BMI, Body mass index; AHI, Apnea-hypopnea index; AI, Apnea index; AHI_supine,_ AHI in a supine position; AHI_REM,_ AHI in a rapid eye movement (REM) stage; AHI_NREM,_ AHI in a non-REM stage; RDI, Respiratory disturbance index; LSaO_2,_ the lowest oxygen saturation; ODI, Oxygen desaturation index; WASO, Wake period after sleep onset; ArTh, the level of arousal threshold; SNA, angle between Sella-Nasion line and Nasion-point A line; SNB, angle between Sella-Nasion line and Nasion-point B line; ANB, A point-Nasion-B point angle; FH, Frankfort plane; ESS, Epworth sleepiness scale; SSS, Stanford sleepiness scale.

When compared by gender, male patients (80.2%) were younger and more obese than females, representing more severe OSA, as indicated by greater AHI (*P* < .01), AI (*P* < .05), AHI_supine_ (*P* < .05), AHI_NREM_ (*P* < .05), and LSaO_2_ (*P* < .05), with lower sleep efficiency (*P* < .05). However, females showed greater skeletal discrepancy in both sagittal (ANB, *P* < .01) and vertical (FH-MP, *P* < .01) aspects than males, presenting negatively lower level of arousal threshold (ArTh, *P* < .01). No gender difference was found in sleepiness symptoms, insomnia index, and the comorbid hypertension and diabetes.

According to age, the elder group over 50 years (51.3%) revealed more severe OSA with greater AHI_supine_ (*P* < .05), AHI_NREM_ (*P* < .05), and LSaO_2_ (*P* < .05) as well as lower sleep efficiency (*P* < .05) and greater WASO (*P* < .05). The prevalence of hypertension (*P* < .001) and diabetes (*P* < .01) were higher in the elder group than in the younger group under 50 years (48.9%).

### Frequency distribution of craniofacial skeletal patterns by gender and age

**Fig 3:**
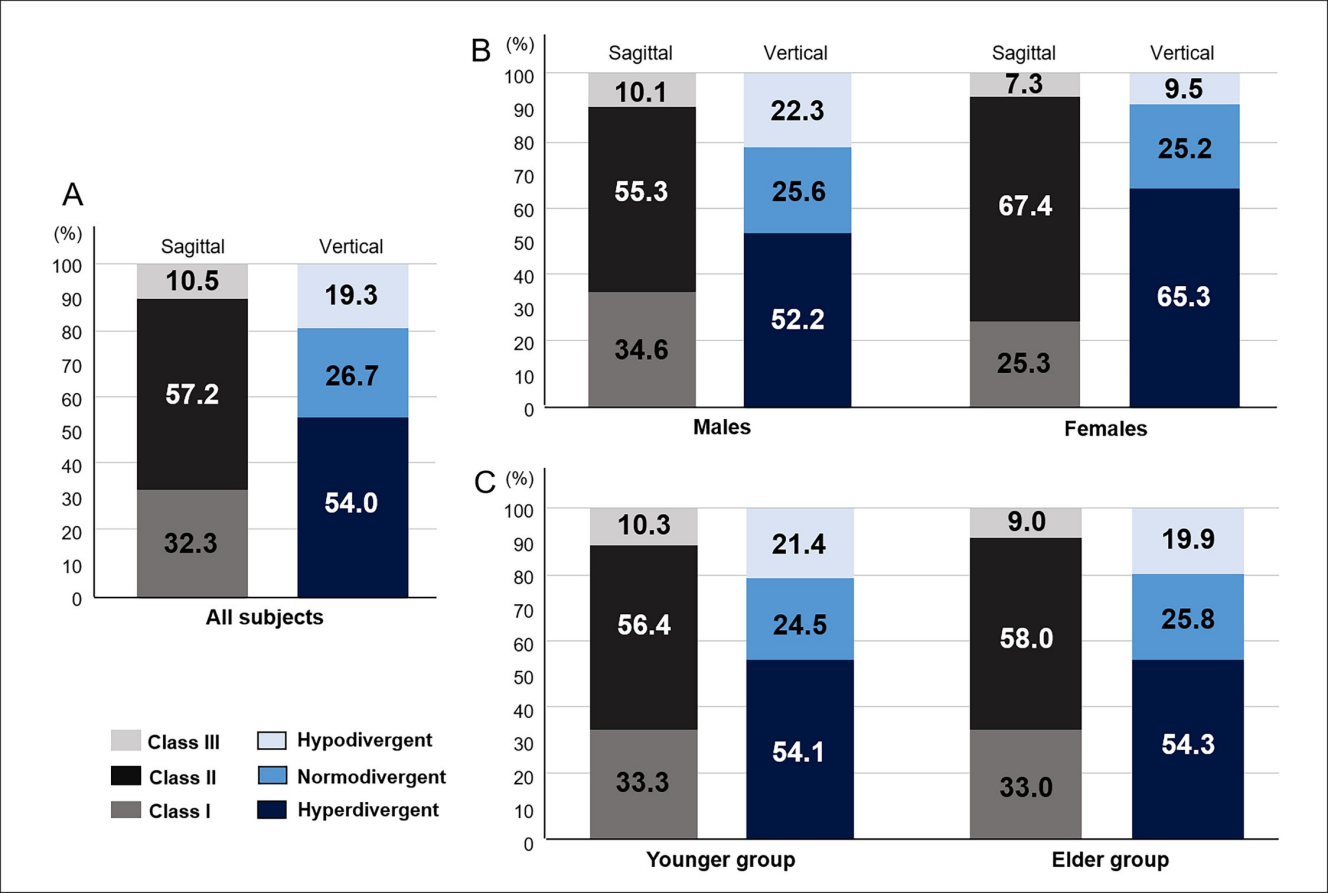
Distribution (%) of sagittal and vertical skeletal patterns in OSA patients (A) by gender (B) and age (C) tested by frequency analysis.

The frequency distribution of the sagittal skeletal classification was 32.3% of Class I, 57.2% of Class II, and 10.5% of Class III, and that of vertical classification was 26.7% of normodivergent, 54.0% of hyperdivergent, and 19.3% of hypodivergent types (Fig 3A). For sagittal pattern, Class II relationship was the most frequent in both males (55.3%) and females (67.4%). For vertical pattern, hyperdivergent pattern was most frequently observed in males (52.2%) and females (65.3%). Females displayed higher frequencies of both Class II and hyperdivergent patterns than males (Fig 3B). The prevalence of hyperdivergent Class II skeletal pattern was 39.1% in total, representing a higher prevalence in females (51.4%) than in males (34.9%). On the other hand, no significant frequency difference in skeletal pattern was observed between the elder and younger groups (Fig 3C).

### Prevalence of skeletal subtypes

According to the independent maxillary and mandibular positions, six skeletal subtypes were obtained (Table 2). The prevalence of mandibular retrusion (56.0%) was the highest. The prevalence of maxillary retrusion and bimaxillary retrusion was 19.3% and 9.1%, respectively. In contrast, maxillary protrusion, mandibular protrusion, and bimaxillary protrusion were observed in 25.1%, 20.1%, and 13.2% of all subjects, respectively. Notedly, mandibular retrusion was highly prevalent in skeletal Class II OSA patients (80.2%), and maxillary retrusion proved to be the key trait of skeletal Class III OSA patients (96.9%)

**Table 2:** Prevalence of sagittal skeletal subtypes in OSA patients.

Dentoskeletal traits	Definition	Overall prevalence (%)	Relative prevalence (%)
Mandibular retrusion	SNB ≤ 77°	56.0	82.0 / Class II
Maxillary protrusion	SNA ≥ 83°	25.1	20.8 / Class II
Mandibular protrusion	SNB ≥ 81°	20.1	65.1 / Class III
Maxillary retrusion	SNA ≤ 79°	19.3	96.9 / Class III
Bimaxillary protrusion	SNA ≥ 83° and SNB ≥ 81°	13.2	-
Bimaxillary retrusion	SNA ≤ 79° and SNB ≤ 77°	9.1	-

SNA, angle between Sella-Nasion line and Nasion-point A line; Normal range, 80≤SNA≤82°.

SNB, angle between Sella-Nasion line and Nasion-point B line; Normal range, 78≤SNB≤80.

### OSA characteristics according to craniofacial skeletal pattern

All tested polysomnographic, symptomatic, and comorbid variables showed no intergroup differences among Class I, Class II, and Class III patterns, as well as among normodivergent, hyperdivergent, and hypodivergent patterns (Table 3). The unique difference was that the patients with skeletal Class III sagittal pattern were more obese (BMI, *P* < .001) than those with Class I or Class II.

**Table 3:** Polysomnographic, symptomatic, and comorbid characteristics of subjects with different craniofacial pattern.

*Variables*	Sagittal skeletal pattern											Vertical skeletal pattern										
	Class I ^a^ (32.3%)			Cass II ^b^ (57.2%)			Class III ^c^ (10.5%)			*P*-value	*Post-hoc comparison*	Normodivergent ^a^ (26.7%)			Hyperdivergent ^b^ (54.0%)			Hypodivergent ^c^ (19.3%)			*P*-value	*Post-hoc comparison*
Age (*year*)	43.4	±	13.2	46.3	±	1.2	38.8	±	7.3	.002**	b>a>c	44.4	±	13.6	44.2	±	15.3	45.8	±	11.8	.091	-
Male (%) ^§^	84.6			72.5			85.3			.013*	-	80.5			76.5			90.1			.026*	-
ANB (°)	2.8	±	1.0	5.9	±	1.2	-1.4	±	0.7	.000***	b>a>c	4	±	1.9	5.7	±	2.0	3.5	±	1.9	.036*	b>a,c
Mandibular plane to FH (°)	24.3	±	4.7	27.2	±	5.2	25.8	±	5.2	.041*	b>a,c	26.1	±	2.1	33.4	±	2.5	19.4	±	2.8	.000***	b>a,c
BMI (*kg/m*^*2*^)	26.4	±	3.9	26.0	±	3.3	29.2	±	4.0	.000***	a,b<c	26.2	±	3.3	26	±	4.6	26.6	±	3.6	.321	-
AHI (*event/hr*)	38.5	±	22.1	40.3	±	23.7	36.9	±	17.9	.482	-	40.4	±	20.3	40.5	±	25.6	39.1	±	20.1	.552	-
AI (*event/hr*)	18.4	±	21.0	19.9	±	21.1	17.4	±	18.2	.078	-	19.2	±	20.5	20.7	±	24.8	19.5	±	19.7	.098	-
AHI_supine_ (*event/hr*)	28.5	±	27.1	31	±	27.8	29.4	±	25.3	.094	-	30.1	±	27.7	30.2	±	29.3	31.1	±	19.7	.052	-
AHI_NREM_ (*event/hr*)	37.8	±	20.1	39.1	±	22.3	39.8	±	18.8	.402	-	39.4	±	21.1	38.6	±	22.2	40.3	±	25.2	.198	-
AHI_REM_ (*event/hr*)	35.5	±	20.5	38.3	±	22.2	34.9	±	21.9	.066	-	34.5	±	22.9	39.5	±	24.6	35.1	±	21.0	.222	-
RDI (*event/hr*)	41.9	±	21.4	43.1	±	23	39.5	±	29.9	.600	-	42.1	±	22.5	43.3	±	24.8	44	±	19.0	.898	-
ODI (*event/hr*)	28.4	±	34.3	29.1	±	23	32.3	±	28.3	.258	-	28.3	±	22.3	28.5	±	25.9	31.9	±	30.1	.066	-
LSaO_2_ (*%*)	82.8	±	8.1	82.3	±	7.8	81.6	±	8.7	.247	-	82	±	8.0	82.5	±	8.9	82.2	±	6.9	.195	-
Sleep efficiency (%)	88.3	±	9.3	86.3	±	8.8	89	±	10.3	.069	-	87.2	±	9.8	86.8	±	8.0	90.2	±	10.5	.092	-
REM (*%*)	18.7	±	6.3	18.4	±	6.6	21.6	±	8.8	.598	-	18.1	±	6.5	18.7	±	7.8	18	±	5.6	.635	-
Arousal index*(event/hr)*	42.6	±	19.5	44.1	±	20.4	45.3	±	26.6	.152	-	43.1	±	20.2	43.5	±	22.7	44.2	±	17.7	.305	-
ArTh (*cm H*_*2*_*O*)	-14.9	±	7.9	-16.4	±	7.5	-16.6	±	9.4	.085	-	-15.3	±	5.5	-17.1	±	8.5	-16	±	8.1	.089	-
ESS (*score*)	9.4	±	4.2	11.5	±	3.5	8.3	±	5.0	.052	-	9.6	±	5.9	10.9	±	4.1	10.2	±	4.7	.096	-
SSS (*score*)	2.9	±	1.1	3	±	1.2	3.4	±	1.2	.252	-	2.9	±	1.2	3.8	±	1.0	3.1	±	1.4	.233	-
WASO (*%*)	11.5	±	15.8	10.2	±	8.3	9.9	±	2.8	.235	-	10.7	±	13.9	10.9	±	11.5	11.2	±	9.6	.100	-
Insomnia (*score*)	10.9	±	6.04	11.7	±	5.4	12.6	±	4.8	.301	-	11.2	±	5.7	11	±	5.0	11.8	±	6.1	.356	-
Hypertension (*%*) ^§^	18.8			31.1			10.2			.021*	-	23.9			25.3			26.0			.630	-
Diabetes (*%*) ^§^	5.5			10.3			6.8			.051	-	5.3			10.8			8.0			.166	-

ANOVA and Scheffe’s post-hoc analysis for continuous variables

^§^, and Pearson’s chi-square tests for categorical variables were performed.

*, P < .0.05

**, P < .0.01

***, P < .0.001. The definition of abbreviation was described in Table 1.

### Skeletal characteristics according to OSA severity

According to OSA severity, 109 patients (8.8%) were mild, 337 patients (27.5%) were moderate, and 780 patients (63.7%) were severe (Table 4). Except for BMI exhibiting the greatest value in the severe OSA group (*P* < .05), all cephalometric and symptomatic variables showed no significant differences according to the OSA severity that was defined by AHI number.

**Table 4:** Symptomatic and cephalometric characteristics of subjects according to OSA severity.

*Variables*	Mild OSA ^a^ (n = 109, 8.8%)			Moderate OSA^b^ (n = 337, 27.5%)			Severe OSA^c^ (n = 780, 63.7%)			*P*-value	*Post-hoc Comparison*
Age (*years*)	41.6	±	10.9	41.5	±	13.3	47.9	±	13.3	.035*	a,b<c
BMI (*Kg/m*^*2*^)	24.1	±	2.2	24.7	±	3.1	27.3	±	3.5	.022*	a,b<c
AHI (*event/hr*)	11.7	±	2.0	22.2	±	4.1	53.7	±	17.2	.000***	a<b<c
ArTh (*cm H*_*2*_*O*)	-15.3	±	5.3	-14.8	±	4.9	-16.8	±	6.8	.065	-
ESS (*score*)	8.6	±	4.2	9.8	±	7.3	11.2	±	4.3	.562	-
SSS (*score*)	2.3	±	0.9	3	±	1.1	3.2	±	1.2	.696	-
Insomnia (*score*)	10.3	±	6.0	12.2	±	6.3	11.8	±	6.1	.520	-
SNA (°)	82.3	±	2.5	81.9	±	2.3	81.5	±	3.2	.356	-
SNB (°)	78.5	±	3.0	77.8	±	3.2	76.5	±	3.9	.068	-
ANB (°)	3.8	±	1.9	4.3	±	2.2	4.4	±	2.0	.055	-
Mandibular plane to FH (°)	25.2	±	4.0	26.4	±	5.5	25.4	±	5.2	.102	-
Facial height ratio (%)	69.9	±	4.6	68.9	±	4.3	69.8	±	4.8	.685	-

ANOVA and Scheffe’s post-hoc analysis were performed

*, P < .0.05

**, P < .0.01

***, P < .0.001. The definition of abbreviation was described in Table 1.

### Prevalence of hyperdivergent skeletal Class II pattern according to OSA characteristics

Univariable regression analysis without adjustment by confounding factors demonstrated that OSA patients with hyperdivergent Class II pattern were more likely to be females (OR 4.52, *P* < .01) and less obese (OR 3.21, *P* < .01) (Table 5). The prevalence of hypertension was also associated with hyperdivergent Class II pattern (OR 2.62, *P* < .05). However, age, AHI, ArTh, ESS were not significantly related to the presence of hyperdivergent Class II pattern.

**Table 5:** Prevalence of hyperdivergent Class II skeletal pattern according to demographic, polysomnographic, and comorbid conditions.

Variables	Category	Total number	Hyperdivergent Class II number (%)	Unadjusted OR (95% CI)
Age (year)	< 50	600	240 (40.0)	1.00
	≥ 50	626	238 (38.0)	0.96 (0.36~1.50)
Gender	Male	983	343 (34.9)	1.00
	Female	243	125 (51.4)	4.52 (3.99~5.91)**
BMI (kg/m^2^)	<18	87	47 (54.0)	3.21 (2.73~4.28)**
	18~25	628	263 (41.9)	1.00
	≥25	511	168 (32.9)	0.90 (0.28~1.81)
AHI (event/hr)	5~15	109	42 (38.5)	1.00
	15~30	337	130 (38.6)	1.02 (0.72~1.51)
	≥30	780	306 (39.2)	1.09 (0.74~1.29)
ArTh (cm H_2_O)	<-15	510	224 (43.9)	1.00
	≥-15	716	254 (35.5)	0.88 (0.69~1.10)
ESS (score)	0~9	201	55 (27.4)	1.00
	9~12	702	301 (42.9)	2.63 (1.88~3.19)
	12~24	323	122 (37.8)	2.13 (1.88~3.19)
Hypertension	No	909	327 (36.0)	1.00
	Yes	317	151 (47.6)	2.62 (1.66~3.01)*
Diabetes	No	1143	445 (38.9)	1.00
	Yes	83	33 (39.8)	1.06 (0.68~1.59)

Univariable regression analysis was performed, OR, odds ratio

*, P < .0.05

**, P < .0.01. The definition of abbreviation was described in Table 1.

## Discussion

This cross-sectional population-based study showed no statistical association between the craniofacial skeletal patterns and OSA presentations assessed by disease severity, symptoms, and sleep quality, although characteristic distribution of craniofacial skeletal patterns existed in OSA patients. To our knowledge, this is the first study to investigate the diverse aspects of sleep and OSA characteristics on the basis of the frequency distribution of various skeletal patterns in a large population of adult patients with OSA. In order of frequency, the distribution of sagittal skeletal pattern was 57.2% of Class II, 32.3% of Class I, and 10.5% of Class III, while that of vertical skeletal pattern was 54.0% of hyperdivergent, 26.7% of normodivergent, and 19.3% of hypodivergent type (Fig 3). As compared to the distribution of skeletal patterns in the healthy Korean population [29], Class II with retruded mandible and hyperdivergent vertical pattern were prevalent in OSA population, corresponding to the previous reports [26–28]. The prevalence of highly risky pattern of hyperdivergent Class II reached 39.1%, on the other hand, maxillary and mandibular protrusive patterns, which might be irrelevant to OSA pathogenesis, accounted for 25.1% and 20.1% of whole samples, respectively (Table 2). The implication of this study is that craniofacial phenotyping based on cephalometric skeletal pattern analysis proved to be insufficient to predict OSA characteristics, not because of no contribution of skeletal factors to OSA risks but because of the highly variable influence of skeletal factors on the OSA characteristics among individuals.

**Fig 4:**
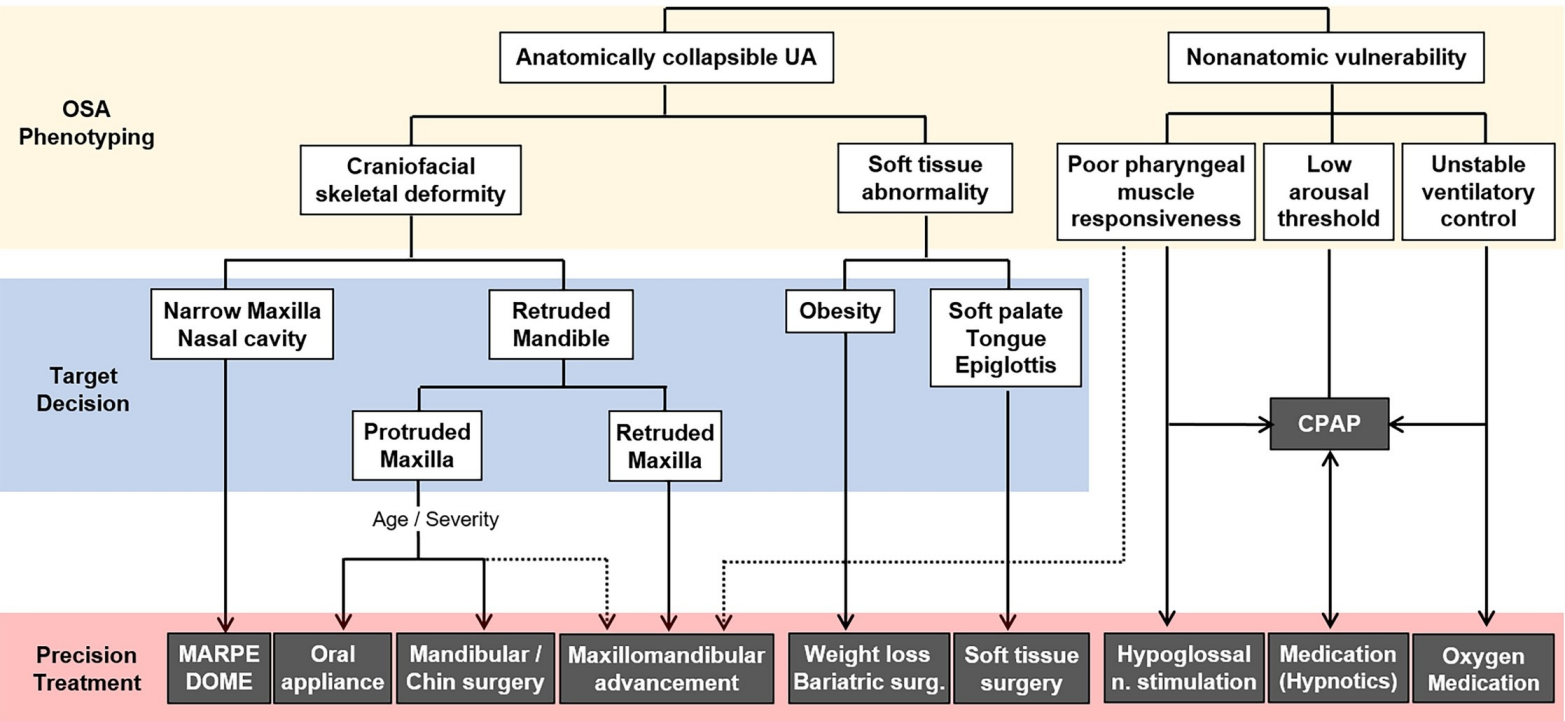
A systematic protocol for phenotype-based precision intervention or combined approach for adult OSA patients. UA, upper airway; MARPE, miniscrew assisted rapid palatal expansion; DOME, Distraction osteogenesis maxillary expansion.

The prevalence of craniofacial skeletal risk factors for OSA varied according to gender and obesity in the present study. There have been few direct inter-gender comparisons of skeletal risk factors for OSA. The unadjusted prevalence of hyperdivergent skeletal Class II pattern in females was 4.52-fold higher than that in males (Table 5). However, female OSA patients who had greater tendency of sagittal and vertical skeletal discrepancies showed significantly less obesity, less OSA severity, higher proportion of AHI_REM_ relative to AHI_NREM_, and lower arousal threshold than male patients, although there existed no gender difference in subjective daytime sleepiness, insomnia, and comorbid hypertension and diabetes. It can be postulated that female OSA patients had higher risk of skeletal contribution to OSA pathogenesis than males, irrelevant to the disease severity and progression. In other aspects, the prevalence of hyperdivergent skeletal Class II pattern was 2.47-fold higher in the underweight group (BMI<18) than in the obese group (BMI≥25). This is consistent with previous studies supporting that nonobese patients displayed more skeletal restriction invading the airway patency whereas obese patients had comparatively larger parapharyngeal soft tissues related to fat deposition with less skeletal restriction [19,30,31], which means different developing mechanism of OSA. Nonetheless, 32.9% of obese patients showed hyperdivergent Class II pattern in the present study, who might have been influenced by synergistic contribution of hard and soft tissue abnormalities to OSA. Accordingly, differential diagnosis among anatomical phenotypic causes, depending on gender difference, is important to determine a relevant therapeutic option out of orthodontic or surgical skeletal reconstruction, soft tissue surgery, weight control, and their combination for OSA patients belong to anatomical phenotype (Fig 4).

On the contrary, there was no significant difference in the prevalence of skeletal risk factors between age groups, although the elder group exhibited higher OSA severity and comorbidity than the younger group. The OSA patients over age 50 years showed higher AHI particularly in a supine position or in a non-REM stage with lower level of LSaO_2_ which may relate to poor pharyngeal muscle responsiveness with age [32,33]. Lower sleep efficiency was noted with longer WASO associated with poor sleep architecture in the elder group. Interestingly, however, anatomical risk factors such as skeletal variables and BMI showed no differences between the two age groups, which implies higher contribution of non-anatomical phenotypic causes to OSA severity and comorbidity in the elderly OSA patients. This finding may explain the limited success rate of skeletal reconstruction treatment like maxillomandibular advancement surgery or mandibular advancement device in the elderly OSA patients even with a skeletal restriction. Therefore, an interdisciplinary combined approach should be primarily considered for the elderly OSA patients.

In addition to hyperdivergent skeletal Class II pattern predisposing to OSA, the present study investigated the frequency distributions of various skeletal subtypes in the OSA population. In sagittal aspect, Class I normal maxillomandibular relationship was present in 32.3%, and skeletal Class III relationship was noted in 10.5%. Most of the Class III OSA patients had maxillary retrusion (96.9%) without or with mandibular protrusion (65.1%), whereas most of Class II OSA patients had mandibular retrusion (82.0%) rather than maxillary protrusion (20.8%). Unexpectedly, the prevalence of bimaxillary protrusion (13.2%) was rather higher than that of bimaxillary retrusion (9.1%) even in OSA patients. In vertical aspects, the prevalence of non-hyperdivergent pattern reached up to 46%. Accordingly, diverse manifestations of skeletal features beyond the risky pattern need to be considered in OSA patients in terms of phenotype-based treatment decision.

More important finding is that there were no significant differences in OSA severity, sleep architectures, and subjective symptoms, among patients with different skeletal patterns (Table 3). All polysomnographic parameters and questionnaire scales indicated no significant differences among Class I, Class II, and Class III, as well as among normodivergent, hyperdivergent, and hypodivergent patterns. Conversely, cephalometric parameters showed no significant differences among mild, moderate, and severe OSA patients (Table 4). Moreover, the unadjusted odds ratio on the prevalence of hyperdivergent skeletal Class II pattern revealed no difference according to the level of AHI, ArTh, or ESS (Table 5). These findings demanded no further search for a prediction model for the predisposition of skeletal characteristics to OSA risk in the present study. In terms of comorbidity, on the other hand, the prevalence of hyperdivergent skeletal Class II pattern was 2.62-fold higher in OSA patients with hypertension than in patients without hypertension. However, this result might be influenced by significant age difference among Class I, Class II, and Class III rather than by direct interaction with skeletal pattern (Table 3).

Dissimilarly to lots of previous studies describing that skeletal Class II with retruded small mandible and hyperdivergent long face were commonly observed in adult OSA patients [24,25], the present study disclosed no statistical association between craniofacial skeletal patterns and OSA characteristics. This discrepancy among studies may be due to (1) racial differences in the baseline anatomical structure [15,34], (2) genetic and epigenetic differences in the prevalence of obesity, skeletal deformity, as well as of OSA [16,23], (3) large interindividual difference despite a single ethnicity. Nonetheless, a recent study reported that Caucasians with less severe anatomical compromise exhibited evidence of a lower ArTh while this was absent in Chinese patients [23], consistent with our finding in Korean patients. The phenotypic causative factors such as increased upper airway collapsibility, long duration of apnea and serious oxygen desaturation related to poor muscle responsiveness, high tendency of blood pressure increase or metabolic disturbance during REM sleep, and the degree of excessive daytime sleepiness and insomnia related to arousal threshold, turned out to have no statistical relations to the anatomical skeletal patterns in the present study. No predictive performance of craniofacial phenotyping for OSA risk or pathogenesis might be attributed to the speculations that (1) a multifactorial pathophysiologic mechanism is more frequently related to adult OSA than isolated simple causative factor [1,35], (2) not all states of anatomically collapsed upper airway by skeletal restriction result in functional disruption of respiration or sleep [28,36]. Based on this, this study highlights the importance of individualized craniofacial phenotyping in the process of OSA phenotyping, therapeutic target decision, and precision intervention or combination approach for each OSA patient. The authors suggest a phenotype-based systematic treatment protocol for adult OSA patients (Fig 4), which will help sleep doctors and dentists intimately collaborate towards a precision management of multifactorial OSA.

This study has following limitations. First, a possibility of referral bias may exist due to the retrospective single-center design. Second, the samples were predominantly male with wide range of age, therefore our findings could not be optimized to render gender-specific or age-specific guideline. Third, out of major phenotypic causes of adult OSA, poor pharyngeal muscle responsiveness and ventilatory instability could not be integrated with the craniofacial anatomical factors to characterize the OSA patients. Lastly, two-dimensional cephalometric analysis could not include the transverse skeletal restriction factor. Nonetheless, this study has strong differential points from the prior studies. Large scale cephalometric measurements for the classification of craniofacial skeletal patterns and subtypes were performed to be matched with the various aspects of functional parameters of OSA. OSA severity was assessed by multiple PSG parameters instead of single AHI number, and indirectly by comorbidities. Sleep quality was evaluated by the proportion of REM sleep, arousal index, and arousal threshold level, assisted by WASO and insomnia severity index. Further study is anticipated to integrate three-dimensional skeletal and upper airway patterns including transverse discrepancy into all categories of phenotypic causes of OSA, to establish a more clinically available guideline for phenotype-based therapeutic approach.

## Conclusions

Skeletal restriction is an important risk factor affecting the upper airway collapsibility mostly in combination with other phenotypic causes. Characteristic distribution of sagittal and vertical skeletal patterns existed in a large population of adult Korean patients with OSA, depending on gender, however no statistical association was found between the skeletal patterns and OSA presentations assessed by disease severity, symptoms, and sleep quality. Although craniofacial phenotyping based on cephalometric skeletal pattern analysis proved to be insufficient to directly predict OSA characteristics due to the large interindividual variation, this study highlights that individualized craniofacial phenotyping needs to be a mandatory process for integrated OSA phenotyping, therapeutic target decision, and precision treatment decision in pursuit of personalized approach for OSA.
